# Textile Foil*Written for the Forty-First Annual Meeting of the Mississippi Valley Dental Association.

**Published:** 1885-05

**Authors:** E. G. Betty


### Textile Foil.*

E. G. BETTY, D.D.S.

This material, I am fully persuaded, has not received at the hands of many operators the attention its merit deserves. I have frequently inquired of others how they like it, how they use it, and what are the results obtained from its use. To these questions a not infrequent reply is—"I know nothing about it. What is it? What do you do with it?" Again, I meet others who use it in daily practice, and their experience with it justifies them in giving it a hearty endorsement; in this I also concur.

When this "foil," if such it can be called, was introduced several years ago, very many were skeptical about it, and listened to the claims made for it with many grains of allowance for the earnestness and enthusiasm with which its inventor heralded its advent.

On the other hand, an operator here and there took hold of it with the avowed determination of giving it an exhaustive trial,

* Written for the Forty-First Annual Meeting of the Mississippi Valley Dental Association.

and subjecting it to such tests as would determine, once for all, whether or no it is worthy of a place upon our very meager list of filling materials.

Since then it has gradually, but surely, won for itself a place that is by no means to be despised, and it is probably safe to say that the time is not far distant when it will be universally regarded as one of the staples, just as is tin foil and, though improperly, amalgam.

The number of materials at our command for filling purposes is so very limited that we can scarcely afford to neglect one that gives such promise of durability and continued usefulness. The ease, thoroughness, and rapidity with which it can be introduced, all recommend its use in a large proportion of cavities that for lack of just such a material would be condemned to amalgam, much to the detriment of the patient and the conscience of the operator. Tin, which was formerly so much in vogue, has almost ceased to be used, and one rarely hears of it now, unless when spoken of in connection with a recent method of combining it with gold for the sake of a therapeutical or chemical effect it was supposed to exert when so used. It may be that this was Dr. Robinson's idea when experimenting to produce the material we now know as textile foil. Possibly he merely intended to revive the use of tin by imparting to it some of the working properties of cohesive gold foil. However that may be, he has certainly done us much service by placing in our hands a weapon with which the amalgam war may be more successfully prosecuted and to a more definite issue, so far as practical results are concerned.

That the foil is all it is claimed to be I am not prepared to say, neither do I believe that it should be given the cold shoulder as it is in the East, possibly for the reason that it is the invention of a backwoodsman, and from Michigan at that.

By the use of this material the conscientious practitioner is often enabled to gracefully extricate himself from many an embarrassing dilemma when confronted by an intelligent patient whose exchequer does not rival the surplus in the National treasury, yet whose ideas upon the subject of amalgam are very pronounced, and not to be trifled with.

It is for these reasons that I wish to call your attention to the subject, and if possible induce those experienced in the manipulation of the material to express their opinion in public, and give their several methods of practice, together with their reasons for so doing. To that end I wish to hazard a few remarks, more in the nature of suggestions than positive statements, and hope they will be productive of a profitable discussion.

There are three classes of cases in which the textile foil may be used with advantage. 1st. Very large simple and compound cavities in bicuspids and molars, where it is desirable to introduce an inexpensive yet reliable permanent filling. 2d. Those cases that would require a large expenditure of time to pack in cohesive gold, to say nothing of the possible detrimental effects of long continued malleting upon the tooth, and where it is desirable to preserve a surface of gold. 3d. For making the cervical border of cavities that extend beyond the gum line, and which are difficult to keep dry sufficiently long to introduce strips or pellets of cohesive gold.

Teeth of the first class alluded to are those that fall a prey to the omnipresent amalgam, mainly for the reason that the operator does not expect, and rarely receives, a commensurate fee for filling with gold. In textile foil he has the wherewith to produce a reasonably priced filling and one that if properly made need not give him cause to blush. A few cylinders of the foil made loosly and of size proportionate to the cavity, are first introduced to form a foundation. The balance of the filling may be made with strips "cut with the grain," and malleted in after the manner of heavy gold; the finishing is to be done as usual. Such a piece of work will serve well for many years and always preserves the original color of the material.

In the second class, the foil plays a not unimportant part, and one that is frequently appreciated by the patient, for the reason that it enables the operator to save much time and expense, and yet produce work that is to all intents and purposes solid gold; I refer to very extensive crown cavities in the molars.

These cavities may be filled one half, and generally two-thirds full of the textile foil well condensed with the mallet, and then covered with a thick superstructure of gold. The latter is better retained in place by large retaining pits drilled into the foil at suitable angles, or an undercut may be left just beneath the enamel, for I do not consider that the so-called "welding" of the gold to the textile material is sufficiently reliable to retain the gold cap firmly in place.

A large filling made in this manner possesses at least one decided advantage over one made exclusively with gold, viz.: it prevents, to a considerable extent, the shock incident upon sudden thermal changes, in this way rivaling oxyphosphate of zinc.

The third class of cases mentioned above, presents many difficulties that are almost insurmountable to the beginner, and not infrequently tax the patience and ingenuity of the most expert, when striving to make a filling of cohesive gold. Very often in such instances, there is not time to promote absorption of the gum with cotton wedges, and the custom of some to dissect away the superimposing soft tissues, not only inflicts unnecessary pain, but produces a hemorrhage, in many instances, that impedes the progress of the work. The cervical border of the cavity, either in bicuspids or molars, is prepared in the usual way, and then covered with cylinders of the foil well malleted into place and brought up just above the free margin of the gum in its normal condition. This can be done quickly, and before moisture has had time to enter the cavity. Once the border is so covered, the case becomes simple, and the gold may then be introduced in perfect confidence of a good result. When first using the textile foil, it was my custom to fill such a cavity, or others of like kind, not extending beyond the gum line, at least half, if not two-thirds full with the foil. Experience has since taught me that the discoloration which ensues when the material is combined with gold, and both metals exposed to the fluids of the mouth, is not near so extensive nor objectionable when the amount of the material is reduced to the least practicable proportion.

Another point in favor of the foil is that it is often much more kindly tolerated in very sensitive teeth than gold, and often better than the oxyphosphates, the acid in the latter, if not prevented in some way, often producing intolerable pain.

